# Beyond prediction: AI as a mechanistic microscope and digital twin for colorectal cancer immunotherapy

**DOI:** 10.3389/fimmu.2026.1746927

**Published:** 2026-06-05

**Authors:** Zijun Zhou, Jianping Zhou

**Affiliations:** 1 Department of Gastrointestinal Surgery, The First Hospital, China Medical University, Shenyang, China; 2 Shenyang Medical Nutrition Clinical Medical Research Center, Shenyang, China

**Keywords:** artificial intelligence, CRC (colorectal cancer), digital twin, immunotherapy, mechanistic microscope

## Abstract

Colorectal cancer (CRC) remains a major cause of cancer-related death, yet the benefits of immune checkpoint inhibitors are limited to a small subset of patients, particularly those with microsatellite instability-high or mismatch repair-deficient tumors. Most patients with microsatellite-stable disease derive little benefit, and even responsive subgroups show substantial heterogeneity and acquired resistance. These challenges highlight the need for biomarkers and therapeutic frameworks that can not only predict response, but also explain underlying biology and support dynamic treatment decisions. In this review, we propose that artificial intelligence (AI) can move beyond prediction to serve two broader roles in CRC immunotherapy: as a mechanistic microscope that reveals hidden tumor–immune interactions from multimodal data, and as a digital twin that models patient-specific therapeutic trajectories over time. We summarize recent advances in AI-based pathology, imaging, and liquid biopsy for pretreatment stratification and response monitoring, and discuss how these approaches may inform resistance mapping, adaptive trial design, and strategies to convert immunologically “cold” tumors into “hot” tumors. We further examine key translational barriers, including generalizability, interpretability, and regulatory validation. By integrating multimodal data with mechanistic modeling, AI may help shift CRC immunotherapy from population-level prediction toward dynamic, individualized precision oncology.

## 1 Introduction

Colorectal cancer (CRC) remains the third most common malignancy worldwide and the second leading cause of cancer-related death (1). Despite advances in screening, surgery, chemotherapy, and targeted therapy, the prognosis of advanced CRC remains poor. Immune checkpoint inhibitors (ICIs) have revolutionized treatment in several cancers, yet their clinical benefit in CRC is largely confined to the 10–15% of tumors with microsatellite instability-high (MSI-H) or mismatch repair-deficient (dMMR) status (2–4). Most microsatellite-stable (MSS) CRC patients remain unresponsive, and even among MSI-H/dMMR tumors, heterogeneous responses and acquired resistance are common (5). Current biomarkers such as MSI, tumor mutational burden (TMB), and PD-L1 expression provide only static snapshots and fail to capture the dynamic complexity of tumor–immune interactions.

Artificial intelligence (AI) offers an unprecedented opportunity to move beyond these limitations. Traditionally viewed as predictive tools, AI systems are now emerging as engines capable of reshaping the framework of CRC immunotherapy. We propose a three-layer paradigm:

Firstly, AI as a Mechanistic Microscope: decoding tumor–immune biology from multimodal data (histology, imaging, liquid biopsy), transforming raw features into mechanistic insight.

Secondly, AI as a Digital Twin: integrating longitudinal pathology, radiomics, and circulating tumor DNA (ctDNA) dynamics into individualized, continuously updated models that simulate therapeutic response and resistance evolution.

Thirdly, AI as a Translational Engine: generating new hypotheses for cold-to-hot tumor transition, informing adaptive clinical trial design, and enabling regulatory-ready pathways for clinical deployment.

Importantly, these three layers should not be viewed as independent modules, but as a sequential translational workflow. In this framework, AI first functions as a mechanistic microscope to identify latent tumor–immune features from multimodal data, such as immune-cell topology, stromal exclusion, clonal dynamics, and ctDNA-derived minimal residual disease signals. These features can then be formalized as patient-specific state variables within a digital twin, enabling dynamic simulation of treatment response, resistance evolution, and therapeutic timing. The resulting simulations can subsequently serve as a translational engine by generating testable hypotheses, prioritizing rational combination strategies, and informing adaptive clinical trial design. This progression from biological decoding to dynamic modeling and finally to clinical hypothesis generation provides the conceptual backbone of the present review.

**Figure 1:**
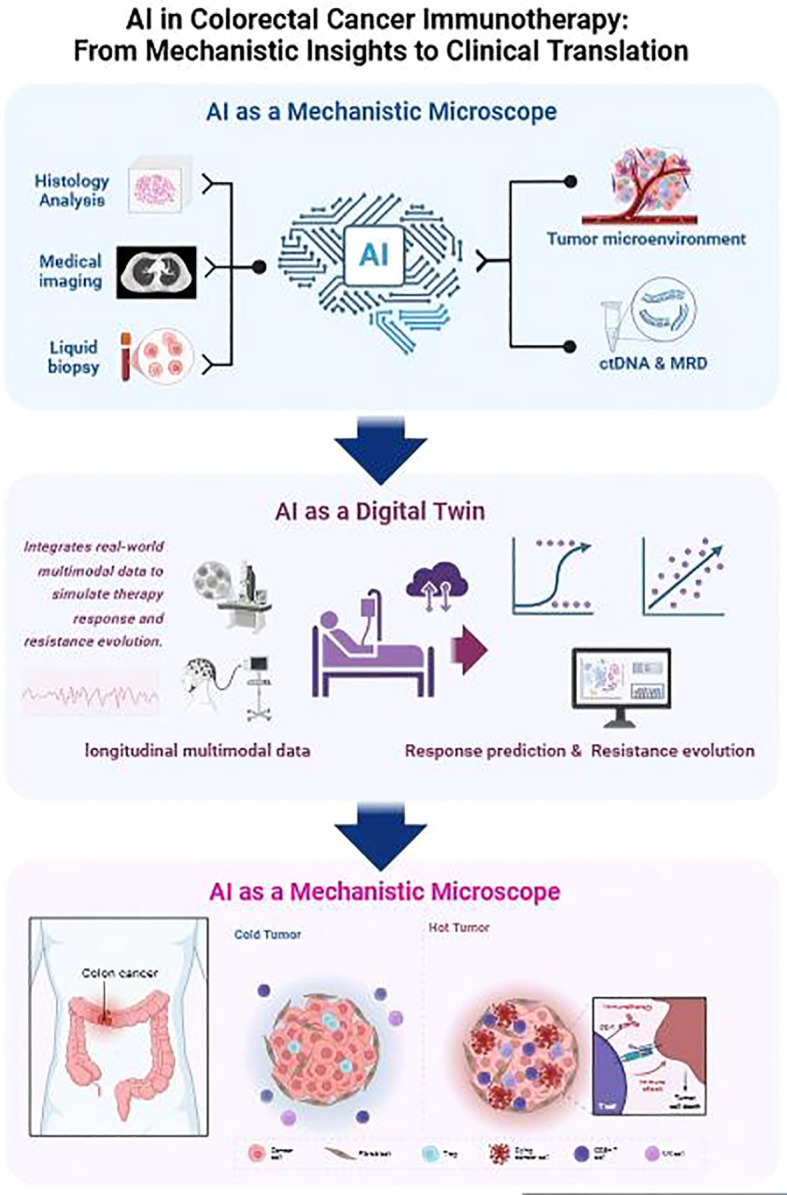
Sequential conceptual framework of AI in colorectal cancer immunotherapy. AI first acts as a mechanistic microscope to extract latent tumor–immune features from multimodal data, then as a digital twin to simulate patient-specific therapeutic trajectories, and finally as a translational engine to generate testable hypotheses, support adaptive trial design, and guide individualized intervention strategies.

Unlike prior reviews that largely discuss AI as a diagnostic or predictive tool. This review synthesizes recent advances in each of these layers, critically examines translational challenges such as generalizability, interpretability, and multicenter validation, and outlines a future in which AI enables a shift from probabilistic medicine to dynamic individualized medicine in CRC immuno-oncology. To our knowledge, this is among the first reviews to organize AI in CRC immunotherapy as a mechanistic-to-translational continuum rather than a set of isolated predictive tools. As illustrated in **Figure 1**, AI functions as a mechanistic microscope, digital twin, and translational engine in CRC immunotherapy.

## 2 Mechanistic microscope: decoding the tumor–immune ecosystem

This section focuses on how AI can uncover hidden features of the tumor–immune ecosystem from multimodal data before or during treatment. Rather than serving only as a prediction tool, AI can help transform pathology, imaging, and liquid biopsy into biologically interpretable signals relevant to immunotherapy response.

AI applied to hematoxylin–eosin (H&E) whole-slide images has enabled direct prediction of key molecular phenotypes in colorectal cancer. Deep learning models can accurately infer microsatellite instability (MSI) or mismatch repair deficiency (dMMR) from histology alone, sometimes matching molecular assays in accuracy (6, 7). Beyond MSI/dMMR, newer multimodal architectures have extended to predicting tumor mutational burden and POLE mutations, broadening the molecular scope of AI-driven pathology (8). At the same time, AI can quantify tumor-infiltrating lymphocytes (TILs) and spatial immune architecture, thereby linking histologic morphology with immune functionality (9). These advances position pathology-based AI as a mechanistic microscope that captures both genotype and immune phenotype from routine slides.

In imaging, radiomics and deep learning models extract quantitative features from CT, MRI, or PET scans to characterize the tumor immune microenvironment. Several studies have demonstrated their ability to distinguish “immune-hot” from “immune-cold” CRCs and to predict therapeutic response in neoadjuvant or immunotherapy settings (10, 11). Meta-analyses confirm that radiomic models can noninvasively predict MSI/dMMR status with clinically meaningful performance (12). Such imaging-based immune phenotyping bridges radiology with systems immunology, providing a complementary macroscopic lens to histopathology.

Liquid biopsy adds a dynamic dimension. AI-enhanced analysis of ctDNA, including fragmentomics, methylation signatures, and mutational spectra, improves the sensitivity and specificity of minimal residual disease (MRD) detection and molecular classification (13). Emerging work also explores AI-assisted profiling of exosomal RNA and proteins to uncover immune-related signatures and identify “hidden beneficiaries” among microsatellite-stable (MSS) patients (14). Collectively, these approaches translate multimodal data—pathology, imaging, and liquid biopsy—into mechanistic information about immune infiltration, clonal evolution, and treatment sensitivity, allowing AI to act as a microscope into the tumor–immune ecosystem.

Collectively, these multimodal AI-derived readouts should be interpreted not merely as static biomarkers, but as mechanistically informative variables that can seed the next analytical layer. Immune-topology patterns from pathology, radiomic signatures of immune exclusion, and liquid-biopsy indicators of clonal evolution or residual disease together provide the state descriptors required for building patient-specific digital twins. In this sense, the mechanistic microscope supplies the biological priors and continuously updated observations that make dynamic simulation clinically meaningful.

Comparative appraisal of current AI methodologies. Pathology-based AI is currently the most mature modality for molecular phenotype prediction in CRC because it builds on routinely available slides and has achieved multicenter external validation in selected studies. However, many models remain retrospective and are vulnerable to stain, scanner, and case-mix biases. Radiomics provides a noninvasive view of whole-tumor heterogeneity, but its reproducibility is often limited by protocol heterogeneity, feature instability, and inconsistent external validation. Liquid-biopsy approaches are attractive for dynamic monitoring because they align more closely with longitudinal disease evolution and clinically meaningful endpoints, yet assay standardization and immunotherapy-specific validation remain underdeveloped. These differences suggest that future AI research in CRC immunotherapy should prioritize prospective benchmarking, multimodal integration, and study designs that assess not only accuracy but also transportability and clinical utility.

## 3 AI-driven digital twins for real-time monitoring

Crucially, digital twins do not operate in isolation from upstream biomarker discovery. Rather, they depend on mechanistically grounded inputs generated by the preceding “microscope” layer, including spatial immune architecture, imaging-defined immune phenotypes, and circulating molecular indicators of tumor burden and immune escape. By converting these observations into dynamic state variables, digital twins move AI from descriptive inference toward temporal prediction and intervention planning.

Dynamic monitoring of treatment response in colorectal cancer is increasingly feasible through longitudinal ctDNA analysis and delta-radiomics modeling. Prospective trials such as CIRCULATE-Japan demonstrated that ctDNA positivity after surgery accurately predicts recurrence and overall survival, supporting its role in minimal residual disease (MRD) tracking and adaptive therapy guidance (15). Beyond ctDNA, delta-radiomics captures temporal changes in tumor phenotype—reflecting microstructural and vascular alterations earlier than conventional size-based criteria—and has shown strong prognostic value in rectal and metastatic CRC (12, 16).

Integration of multimodal data—including histopathology, imaging, ctDNA, and clinical variables—forms the foundation of individualized digital twins. These computational replicas continuously assimilate real-world data to simulate treatment efficacy, resistance evolution, and optimal therapeutic pathways (17). Quantitative systems pharmacology (QSP) models provide the mechanistic backbone for simulating tumor–immune interactions, while machine-learning algorithms enable real-time parameter updating (18). This synergy transforms CRC management from reactive monitoring to proactive simulation—each patient having a “virtual counterpart” that allows in silico rehearsal of therapeutic strategies before clinical implementation (19). Core components of AI-driven digital twins are shown in **Table 1**.

**Table 1:** Core components of AI-driven digital twin frameworks for CRC.

Layer	Data source	Modeling approach	Clinical function	Representative study
Molecular Layer	ctDNA, exosomal RNA	Bayesian + ML integration	MRD tracking	CIRCULATE- Japan (2022) (15)
Imaging Layer	CT/MRI delta-radiomics	Deep CNN temporal analysis	Dynamic tumor monitoring	(16)
Mechanistic Layer	Quantitative Systems Pharmacology	ODE/agent-based modeling	Simulate immune dynamics	(17, 18)
Integration Layer	Multimodal fusion	Reinforcement learning	Adaptive therapy planning	(19)

## 4 Translational loop – AI as a hypothesis generator and trial engine

At the translational stage, the goal is no longer only to detect biological patterns or simulate disease trajectories, but to convert these mechanistic and dynamic insights into actionable clinical hypotheses. In other words, the translational engine represents the downstream extension of the microscope–digital twin workflow, where decoded tumor–immune mechanisms and simulated response trajectories are used to prioritize interventions, design adaptive trials, and define biomarker-guided treatment strategies.

### 4.1 Tumor immune microenvironment

This subsection examines how AI can translate spatial tissue patterns into clinically interpretable maps of the tumor immune microenvironment. AI on whole-slide images can map TIL density and spatial organization, revealing immune-excluded vs infiltrated patterns associated with response/resistance across tumor types, including CRC (9). In CRC specifically, high-resolution multiplex/spatial studies uncovered coordinated cellular neighborhoods and immune hubs at the invasive front that orchestrate antitumor immunity—providing mechanistic substrates that AI can quantify at scale (4, 20).

Coupling spatial transcriptomics with deep learning further enables cell-type deconvolution and ligand-receptor context mapping from tissue sections. Methods like Tangram (DL alignment of sc/snRNA-seq to spatial data) and cell2location (Bayesian cell-type mapping) allow integrative TIME atlases that AI can mine for immune-escape circuits and targetable niches in CRC (21, 22).

Recent advances in spatial transcriptomics and spatially resolved multi-omics further expand this direction. Beyond cell-type mapping alone, newer AI-enabled frameworks increasingly integrate transcriptomic, proteomic, imaging, and histologic information to reconstruct tissue ecosystems with higher functional resolution. Although many of these approaches were first developed in broader oncology settings rather than specifically in CRC, they are highly relevant to CRC immunotherapy because they improve characterization of immune exclusion, stromal organization, and cell–cell communication. These methods may therefore strengthen the mechanistic microscope layer and provide richer state variables for future digital twin models, although CRC-specific translational validation remains limited (23–25).

### 4.2 Clonal evolution and resistance pathways

This subsection focuses on how AI can reconstruct resistance as an evolving process rather than a single end point. For resistance that emerges under therapy, ctDNA + machine learning can reconstruct clonal architectures and track subclonal dynamics over time. In CRC, clinical reviews detail how longitudinal liquid biopsy supports tumor evolution tracking and therapy decisions (e.g., anti-EGFR rechallenge) (26). Bayesian/ML frameworks like PyClone infer clonal populations from sequencing data, forming the backbone for AI-assisted phylogenies that link emerging clones to resistance phenotypes (27).

In practice, ctDNA-driven strategies have already been used to time anti-EGFR rechallenge in metastatic CRC, and umbrella reviews summarize ongoing trials and algorithms that operationalize these decisions—approaches that can be generalized to immunotherapy resistance modeling within digital twins (28).

### 4.3 Cold-to-hot tumor transition hypotheses

This subsection addresses one of the most clinically relevant uses of the framework: generating testable strategies to convert immune-cold tumors into immune-responsive states. TIME-aware AI (histology + spatial omics) can generate testable hypotheses for converting “cold” MSS CRC into “hot” immune-reactive tumors—e.g., identifying stromal barriers, suppressive myeloid neighborhoods, or T-cell–excluded architectures as intervention targets (20). Contemporary reviews synthesize combination strategies (IO + chemotherapy/radiotherapy/targeted agents) specifically for MSS CRC, outlining mechanistic rationales and biomarkers that AI can operationalize into patient-level treatment simulations (29, 30). Together, these tools support a learning loop where AI detects escape, infers clonal/TIME mechanisms, and proposes cold-to-hot conversions to be tested in translational studies and trials. Translational AI applications across biological levels are outlined in **Table 2**.

**Table 2:** AI-enabled hypothesis generation across translational levels.

Translational level	AI application	Biological/clinical insight	Potential impact
Tissue (Histology + Spatial)	TIME mapping via deep learning	Identify immune-excluded zones	Guide cold-to-hot transition strategies
Liquid	ctDNA + ML clonal tracking	Detect emerging resistant clones	Enable adaptive therapy design
Systems	Digital Twin simulation	Predict synergistic IO combinations	Accelerate in silico trial development

A representative example of this three-layer workflow is the AI-guided “cold-to-hot” transition hypothesis. At the mechanistic microscope level, multimodal AI may identify an immune-excluded phenotype characterized by stromal barriers, sparse intratumoral T-cell infiltration, suppressive myeloid enrichment, and ctDNA evidence of persistent resistant clones. These features can then be encoded within a digital twin to simulate whether specific interventions—such as anti-angiogenic therapy, radiotherapy, or myeloid-targeting combinations—might improve immune infiltration or sensitize the tumor to checkpoint blockade over time. The translational engine then uses these simulation-informed outputs to generate testable clinical hypotheses, for example by prioritizing biomarker-enriched combination regimens or adaptive trial arms for patients with immune-cold MSS CRC. This type of end-to-end workflow illustrates how AI can connect biological decoding to therapeutic experimentation.

## 5 Translational challenges and clinical integration

Before AI models can influence real-world CRC immunotherapy decisions, they must be embedded within existing clinical workflows rather than evaluated as stand-alone algorithms. In practice, this means that AI outputs need to enter the care pathway at defined decision points—for example, prescreening MSI status from pathology slides, stratifying immune phenotypes from imaging, monitoring molecular residual disease through ctDNA, or supporting multidisciplinary review when treatment resistance is suspected. At each of these steps, practical adoption depends not only on model performance, but also on interoperability with laboratory and hospital information systems, clinician interpretability, validation across institutions, and regulatory compliance. The following challenges should therefore be understood as workflow barriers to implementation rather than purely technical limitations.

### 5.1 Generalizability

AI models in digital pathology and radiomics often exhibit site-specific bias. Histopathologic models trained on slides from a single lab frequently lose accuracy when applied to data with different stains, scanners, or pre-analytical conditions (31). Deep-learning–based stain normalization and domain adaptation methods improve cross-site performance but cannot completely eliminate distribution shift (32). Similar challenges exist in radiomics, where imaging protocol differences and scanner heterogeneity limit reproducibility. Recent harmonization frameworks, such as ComBat and deep-learning–based CT normalization, show promise for standardizing quantitative features across institutions (33, 34). Clinically, without robust cross-site generalizability, even high-performing models may fail at the point of care and limit equitable deployment.

### 5.2 Explainability and clinical trust

A key barrier to adoption is explainability. Systematic reviews show that explainable AI (XAI) techniques—such as saliency mapping and feature attribution—can enhance clinicians’ trust, provided the outputs are concise, interpretable, and aligned with pathology or imaging findings (35). However, XAI must be embedded within validated clinical workflows, with user-centered evaluation to ensure safety and accountability in real-world practice. Thus, explainability is not only a technical preference, but a prerequisite for clinician confidence and responsible adoption.

### 5.3 Validation and evidence gaps

Despite impressive technical performance, few CRC AI models have undergone prospective, multicenter validation. A 2024 meta-analysis of diagnostic AI in digital pathology highlighted the lack of large-scale, real-world trials as a major limitation to clinical translation (36). Even advanced CRC models like Deepath-MSI emphasize the need for prospective external validation before regulatory or routine use (37). From a translational perspective, prospective multicenter evidence is essential if AI systems are to move from promising prototypes to trusted decision-support tools.

### 5.4 Regulatory pathways

The approval of MSIntuit-CRC as a CE-marked prescreening AI tool for MSI detection in Europe illustrates the growing maturity of regulatory frameworks. However, developers must carefully distinguish between research-use-only (RUO) and certified diagnostic claims, citing regulatory status explicitly “per manufacturer communication” rather than inferring approval from peer-reviewed studies (38). Broader analyses of AI regulation in the European Union underscore the need for transparent validation, data governance, and post-market monitoring under CE-IVDR guidelines (39). In this sense, regulatory readiness should be considered a core component of clinical translation rather than a downstream administrative step.

### 5.5 Human–AI collaboration and quality assurance

Successful deployment depends on multidisciplinary teamwork and quality control. The College of American Pathologists (CAP) recommends lab-specific validation and revalidation after substantial workflow or software changes (40). Digital pathology QA initiatives further emphasize end-to-end quality assurance across scanning, inference, and reporting to prevent error propagation and overreliance on automation (41). Institutions should maintain model version logs, monitor drift, and require multidisciplinary team (MDT) review for AI-influenced decisions to ensure patient safety and accountability. Emerging solutions to translational and regulatory barriers are listed in **Table 3**. Ultimately, sustainable adoption will depend not only on model accuracy, but also on governance, workflow integration, and accountability.

**Table 3:** Translational and regulatory barriers with emerging solutions.

Category	Core issue	Illustrative example	Emerging solutions
Generalizability	Cross-site domain bias	H&E staining variation	Domain adaptation; federated learning
Explainability	Black-box outputs	Misaligned saliency maps	XAI with clinician co-design
Validation	Lack of prospective trials	DeepPath-MSI pending validation	Multicenter registry frameworks
Regulatory	RUO vs CE-IVD ambiguity	MSIntuit-CRC case	Transparent labeling and traceability
Quality Assurance	Model drift and unlogged updates	Lack of version tracking	CAP re-validation; MDT oversight

## 6 Future outlook – from prediction to paradigm shift

In the short term, AI will continue to serve as a biomarker-assistive tool, augmenting conventional diagnostics and therapeutic decision-making. Deep-learning pathology and radiomics models are already showing clinical utility in predicting microsatellite instability, immune phenotypes, and minimal residual disease—transforming routine data into actionable biomarkers (13, 37).

In the medium term, the integration of AI and quantitative systems pharmacology is expected to accelerate the emergence of digital twin–based clinical research. Early implementations of in silico trial arms—virtual cohorts that simulate patient outcomes under different treatment strategies—have demonstrated feasibility for refining study design, identifying responder subgroups, and optimizing combination therapy strategies in immuno-oncology (17, 42). These developments align with the growing adoption of model-informed drug development (MIDD) frameworks by regulatory agencies, which recognize digital simulation as a valid adjunct to early-phase clinical testing (39).

In the long term, colorectal cancer immunotherapy is poised to evolve from probabilistic medicine to dynamic individualized medicine. AI-driven digital twins could continuously learn from real-time clinical data, anticipate resistance, and adapt therapeutic regimens dynamically under multidisciplinary oversight (18).

Taken together, these developments suggest that AI may gradually shift CRC immunotherapy from static prediction toward mechanism-informed and dynamically adaptive care. By merging mechanistic modeling, multimodal data integration, and adaptive learning, AI holds the potential to turn each CRC patient into a continuously learning system, closing the loop between prediction, experimentation, and personalized care.

## 7 Prioritized roadmap for research and translation

Based on the evidence and gaps synthesized above, a practical research agenda for AI in CRC immunotherapy should be prioritized across three stages. First, near-term efforts should focus on robust benchmarking and external validation of pathology-, radiomics-, and liquid-biopsy–based models across multicenter cohorts, with standardized reporting of calibration, transportability, and clinically relevant endpoints rather than discrimination metrics alone. This priority follows directly from the current evidence base, in which many promising models remain retrospective and vulnerable to domain shift, protocol heterogeneity, and assay variability.

Second, medium-term work should emphasize multimodal integration and mechanism-aware modeling. Rather than continuing to develop isolated predictors for single modalities, future studies should integrate histology, imaging, ctDNA, and emerging spatial or multi-omics readouts into unified patient-level representations. This would enable digital twin models to move beyond conceptual promise toward quantitatively grounded simulation of response, resistance evolution, and treatment timing.

Third, longer-term translation should target prospective clinical implementation. This includes embedding AI outputs into multidisciplinary workflows, testing biomarker-guided and simulation-informed strategies in adaptive trials, and aligning model development with regulatory and quality-assurance requirements from the outset. In practice, lower-risk applications such as MSI prescreening, MRD monitoring, and workflow triage are likely to provide the most realistic entry points, while higher-risk treatment recommendation systems will require substantially stronger prospective evidence.

Taken together, this roadmap suggests that progress in CRC immunotherapy will depend less on developing ever more predictive algorithms in isolation and more on building validated, multimodal, and clinically governable systems that connect mechanistic decoding to real-world decision support. This priority is further supported by recent systematic evidence from blood-based CRC biomarker studies, in which small sample sizes, potential selection bias, limited cost-effectiveness assessment, and insufficient multicenter prospective validation remain major barriers to clinical translation (43).

## 8 Convergence and implementation – toward an AI-enabled precision oncology ecosystem

For AI to move from a prediction aid to a clinically useful infrastructure, three elements must converge: data architecture, clinical integration, and regulatory lifecycle design.

At the data architecture level, federated learning has emerged as a key pathway for collaborative modeling that enables multiple institutions to train algorithms jointly without sharing raw data—balancing privacy preservation with model generalizability. Recent reviews have highlighted practical challenges such as non-independent data distributions, communication costs, and model alignment difficulties, underscoring the need for harmonized standards across medical centers (44, 45). Advances in bias mitigation and fairness-aware algorithms within federated learning frameworks further enhance model equity and transparency in healthcare (46). To support mechanistic interpretability and cross-institutional interoperability, developing dynamic ontologies and unified data schemas is essential—linking pathology, radiomics, genomics, and circulating tumor DNA (ctDNA) features across diverse healthcare ecosystems.

At the clinical integration level, AI modules should evolve from static predictors into continuously learning decision-support systems embedded within multidisciplinary tumor boards. Integration of AI outputs into electronic health records (EHRs), imaging archives (PACS), and biobanks can establish a feedback loop in which virtual simulations inform therapeutic strategies, and real-world clinical outcomes iteratively refine the digital twin models. This dynamic coupling of “virtual” and “real” care exemplifies the core mechanism of precision oncology in the era of adaptive immunotherapy.

Illustrative clinical integration scenario. A practical use case can be envisioned in a patient with MSS colorectal cancer undergoing evaluation for systemic therapy. At diagnosis, a pathology-based AI model could prescreen routine H&E slides for immune-excluded architecture and flag features associated with poor likelihood of response to checkpoint inhibitor monotherapy. In parallel, radiomics analysis from baseline CT or MRI could characterize whole-lesion heterogeneity and stromal exclusion, while ctDNA profiling could establish a baseline measure of molecular burden and clonal composition. These outputs would not act as stand-alone decisions, but would be integrated into the multidisciplinary tumor board through the electronic health record and imaging archive. A patient-specific digital twin could then combine these multimodal inputs to simulate potential treatment trajectories—for example, whether a combination strategy aimed at overcoming immune exclusion might be more rational than standard immunotherapy alone. During follow-up, repeat imaging and ctDNA measurements could update the model, allowing clinicians to detect emerging resistance earlier and modify therapy accordingly. In this scenario, AI serves not as an autonomous decision-maker, but as a continuously updating decision-support layer embedded within routine pathology, radiology, molecular monitoring, and MDT review.

At the regulatory and translational level, global agencies have accelerated formal guidance on AI-enabled device software functions. The US Food and Drug Administration (FDA) released its Final Guidance on Predetermined Change Control Plans (PCCPs) in December 2024, outlining structured mechanisms for post-market algorithm updates (47). The accompanying Lifecycle Management and Marketing Submission Recommendations draft further details risk stratification, transparency, and pre-approval processes to maintain accountability across iterative model revisions (48). These frameworks, together with ongoing European CE-IVDR adaptation, represent a paradigm shift toward “compliance by design” for adaptive AI systems in medicine (49). In practical terms, lower-risk use cases such as MSI prescreening or workflow triage may reach deployment earlier than higher-risk systems that directly recommend treatment changes. This staged adoption pathway may be more realistic for CRC immunotherapy than attempting immediate end-to-end automation.

Ultimately, a true AI-enabled precision oncology ecosystem would integrate federated multimodal data, continuous clinical learning, and regulatory governance into a single translational loop. Within this system, digital twins would evolve from theoretical constructs into actionable bridges linking biological mechanisms, patient data, and therapeutic intent—closing the gap between simulation and treatment in colorectal cancer immunotherapy. However, achieving this ecosystem in practice will require more than technical feasibility. Hospitals will need standardized data pipelines, local IT support for integration with EHR/PACS/LIS systems, clinician training, prospective governance for model updates, and reimbursement or institutional incentives that justify adoption. Without these operational supports, even well-validated AI models may remain confined to pilot studies rather than routine care.

## 9 Conclusion

AI may reshape colorectal cancer immunotherapy in three connected ways: by revealing hidden tumor–immune biology, by modeling patient-specific treatment trajectories, and by generating clinically testable therapeutic hypotheses. Together, these roles outline a framework that moves beyond static biomarkers toward more dynamic and mechanism-informed care.

The next challenge is not only to improve algorithmic performance, but also to establish rigorous validation, trustworthy clinical integration, and clear regulatory pathways. If these barriers can be addressed, AI could help make CRC immunotherapy more individualized, adaptive, and clinically actionable.
